# Establishment of a prognostic model based on m^6^A regulatory factors and stemness of hepatocellular carcinoma using RNA-seq data and scRNA-seq data

**DOI:** 10.1007/s00432-023-05045-x

**Published:** 2023-07-19

**Authors:** Yan Liang, Sen Chen, Jinghe Xie, Guanrong Yan, Tingting Guo, Tianyang Li, Shoupei Liu, Weiping Zeng, Shuai Zhang, Keqiang Ma, Honglin Chen, Yimeng Ou, Bailin Wang, Weili Gu, Yuyou Duan

**Affiliations:** 1 Laboratory of Stem Cells and Translational Medicine, School of Medicine, South China University of Technology, Guangzhou, 510006 People’s Republic of China; 2 Laboratory of Stem Cells and Translational Medicine, Institute for Medical Research, The Second Affiliated Hospital, School of Medicine, South China University of Technology, Guangzhou, 510180 China; 3 School of Biomedical Sciences and Engineering, Guangzhou International Campus, South China University of Technology, Guangzhou, 510006 People’s Republic of China; 4 Department of Gastroenterology and Hepatology, Guangzhou Digestive Disease Center, School of Medicine, Guangzhou First People’s Hospital, South China University of Technology, No.1 Panfu Road, Guangzhou, 510180 People’s Republic of China; 5 School of Biology and Biological Engineering, South China University of Technology, Guangzhou, 510180 China; 6 Department of Hepatobiliary Pancreatic Surgery, Huadu District People’s Hospital of Guangzhou, Guangzhou, 510800 People’s Republic of China; 7 Department of General Surgery, The First Affiliated Hospital of Guangdong Pharmaceutical University, 19 Nonglin Xia Road, Guangzhou, 510699 People’s Republic of China; 8 Department of General Surgery, Guangzhou Red Cross Hospital, Jinan University, 601 Huangpu Dadao West Road, Guangzhou, 510220 People’s Republic of China; 9 National Engineering Research Center for Tissue Restoration and Reconstruction, South China University of Technology, Guangzhou, 510180 China; 10 Laboratory of Stem Cells and Translational Medicine, Institute for Clinical Medicine, The Second Affiliated Hospital, School of Medicine, South China University of Technology, No.1 Panfu Road, Guangzhou, 510180 People’s Republic of China

**Keywords:** HCC, m^6^A modification, CSCs, Stemness, Prognostic model

## Abstract

**Background:**

Hepatocellular carcinoma (HCC) with high incidence and mortality is one of the most common malignant cancers worldwide. Increasing evidence has reported that N6-methyladenosine (m^6^A) modification has been considered as a major contribution to the occurrence and development of tumors.

**Method:**

In our study, we comprehensively analyzed the connection between m^6^A regulatory factors and cancer stem cells (CSCs) of HCC to establish a clinical tool for predicting its outcome. First, we concluded that the expression level of m^6^A regulatory factors was related with the stemness of hepatocellular carcinoma. Subsequently, we gained a ten hub regulatory factors that were associated with prognosis of hepatocellular carcinoma by overall survival (OS) analysis using ICGC and TCGA datasets, and these regulatory factors included YTHDF1, IGF2BP1, METTL3, IGF2BP3, HNRNPA2B1, IGF2BP2, RBM15B, HNRNPC, RBMX, and LRPPR. Next, we found that these ten hub m^6^A regulatory factors were highly expressed in CSCs, and CSCs related pathways were also enriched by the gene set variation analysis (GSVA). Then, correlation, consensus clustering and PCA analysis were performed to reveal potential therapeutic benefits of HCC. Moreover, univariate Cox regression (UNICOX), LASSON and multivariate Cox regression (MULTICOX) analyses were adopted to establish HCC prognosis prediction signature.

**Results:**

Four regulatory factors RBM15B, LRPPRC, IGF2BP1, and IGF2BP3 were picked as valuable prognostic indicators.

**Conclusion:**

In summary, these ten hub regulatory factors would be useful therapeutic targets for HCC treatment, and RBM15B/LRPPRC/IGF2BP1/IGF2BP3 prognostic indicators can be used to guide therapy for HCC patients.

**Supplementary Information:**

The online version contains supplementary material available at 10.1007/s00432-023-05045-x.

## Introduction

The major histological subtype of liver cancer is HCC which accounted for 80–90% of primary liver cancer and the third most common reason for tumor-related mortality worldwide (Pan et al. 2011; Yang et al. 2019). Both high recurrence and metastasis rates contribute to the poor prognosis of HCC (Siegel et al. 2021). In clinical, transplantation of liver is the only treatment for terminal cancer, 5-year survival was gained by about 60%, but due to the limited donor, surgical removal of the lesions and transcatheter hepatic artery embolization with conventional chemotherapy treatment is the main choice of clinical treatment (Alqahtani, et al. 2019). It is necessary to establish a scoring model for the prognosis of HCC patients to provide the theoretical basis for the treatment selection and prognostic evaluation of clinicians.m^6^A RNA modification, the most dominant RNA modification, has been shown to play a crucial role in regulating RNA metabolism (Yang et al. 2018). m^6^A regulatory factors consists of “writers” that generate the m^6^A markers, “erasers” that can remove the m^6^A modification that has occurred, and “readers” that identify the m^6^A modification and further promote the transcription of downstream genes. m^6^A modification affects RNA metabolism in many ways, such as processing, nuclear output, translation, and decay of RNA (Yang et al. 2019; Wang et al. 2021; Shi et al. 2017; Roignant and Soller 2017). m^6^A RNA methylation plays many roles in cancer through a variety of mechanisms and offers more possibilities for early diagnosis and treatment of cancer. In addition, m^6^A modification also plays a critical role in CSCs (Wang et al. 2021). m^6^A RNA modification has been certified to be involved in the production and maintenance of CSCs, control of tumor progression and treatment resistance (Yang et al. 2018; Roignant and Soller 2017; Corbett 2018). In recent years, genetic signatures of m^6^A regulatory factors have been constructed to predict the prognosis of a variety of tumors, including gastric cancer (Guan et al. 2020), adrenocortical carcinoma (Jin et al. 2021), and liver cancer (Li, et al. 2021a). However, it has not been clarified the exact association between m^6^A and CSCs of HCC.

Giving the RNA-seq data available online, we tried to analyze whether the expression patterns of m^6^A regulatory factors can predict prognosis in HCC patients. Tathiane M. Malta et al. used an innovative single-class logistic regression (OCLR) machine learning algorithm to generate mRNAsi index that reflects the gene expression characteristics of stem cells through multi-platform analysis of stem cell transcriptome, methylation and transcription factor binding sites (Malta et al. 2018). The stemness index based on mRNA expression (mRNAsi index) is a measure of how similar tumor cells to stem cells, the higher the mRNAsi index, the stronger the stemness of tissues is. For understanding the association between m^6^A regulatory factors expression and tissue stemness, we accorded to the median data of mRNAsi index and split HCC patients into two groups with high-mRNAsi and low-mRNAsi, respectively, using TCGA (The Cancer Genome Atlas) dataset. In addition, we collected 23 m^6^A regulatory factors in this study and selected m^6^A differential regulatory factors (DEGs) from patients with high-mRNAsi and low-mRNAsi for further study in TCGA dataset. Furthermore, we selected m^6^A DEGs that had prognostic value to be candidate genes in both TCGA and ICGC (The International Cancer Genome Consortium). We found that these hub m^6^A regulatory factors were highly expressed in liver cancer stem cells (LCSCs) using scRNA-seq data (GSE149614) and RT-qPCR. GSVA analysis also showed that the hub m^6^A regulatory factors’ expression was associated with the CSCs related pathways. Moreover, we performed the analyses of correlation, function, Consensus, PCA, survival, and ROC to investigate the biological functions and mechanisms of these regulatory factors in HCC patients, highlighting the importance of these candidate hub regulatory factors. In addition, we observed the prognostic role of m^6^A regulatory genes in HCC, and build a prognostic model in ICGC, and verified it in TCGA. Finally, the overall survival of HCC patients could be better estimated using the prognostic model. In summary, our work suggests that the m^6^A regulatory factors are a potential prognostic indicator for HCC patients.

## Materials and methods

### HCC data source and preprocessing

The data of gene expression data and clinical annotations were gained from ICGC and TCGA datasets. Two cohorts were enrolled in our work: TCGA-LIHC (371 samples) and ICGC-LIRI-JP (243 samples). For mRNA expression data, FPKM-normalized, log2-transformed data were gained, respectively, from web sites Genomic Data Commons Data Portal (GDC Data Portal) (RRID: SCR_014514), ICGC Data Portal (RRID: SCR_021722). Clinical annotations in ICGC with 232 HCC patients and in TCGA with 240 HCC patients were also downloaded for further analysis. The data of Copy number variation (CNV) were gained from TCGA. In addition, the scRNA-seq (GSE149614) of HCC was downloaded from Gene Expression Omnibus (GEO) (RRID: SCR_005012) (Zheng et al. 2018).

23 m^6^A regulatory factors were identified from PubMed (RRID: SCR_004846), including 8 writers (METTL3, METTL14, WTAP, METTL16, RBM15B, ZC3H13 and VIRMA) (Roundtree et al. 2017; Chen et al. 2019), 3 erasers (ALKBH5, ALKBH7 and FTO) (Jia et al. 2011; Zheng et al. 2013), and 12 readers (IGF2BP1, IGF2BP2, IGF2BP3, YTHDF1, YTHDF2, YTHDF3, HNRNPA2B1, YTHDC1, YTHDC2, HNRNPC, LRPPRC and RBMX) (Chen et al. 2019; Du et al. 2021; Casella et al. 2019). We gained the gene expression matrix of corresponding m^6^A regulatory factors from TCGA and ICGC data for further investigation.

### Identification of m^6^A regulatory factors CNV and expression difference between high-mRNAsi and low-mRNAsi groups

The m^6^A regulatory factors expression matrix and CNV data were applicated to the differentially CNV genes and the DEGs between high-mRNAsi and low-mRNAsi patients. According to the median data, we first classified HCC patients into high-mRNAsi and low-mRNAsi group to identify among the 371 samples in TCGA. Then R package “limma” was used to determine m^6^A DEGs by comparing high-mRNAsi specimens with low-mRNAsi specimens. 20 m^6^A DEGs (adjusted *P* < 0.05) in TCGA were identified for further study.

### The Kaplan–Meier curve based on m^6^A DEGs in HCC patients

Among 17 m^6^A differential regulatory factors in TCGA and 13 m^6^A regulatory factors DEGs in ICGC, we figured out the potential prognostic m^6^A differential regulatory factors by Survival analysis and Kaplan–Meier curves using the “survival” R package (adjusted *p* < 0.05). The *R* package “survminer” and a two-sided log-rank test were used to identify the optimal cutoff value. To gain more accurate results, a Venn plot was used to obtain the overlapped prognostic m^6^A different regulatory factors in either TCGA or ICGC. Finally, ten shared hub regulatory factors were left for further analysis.

### scRNA-seq data analyses

scRNA sequence data of different sites of HCC was downloaded, including ten HCC patients from four relevant sites: primary tumor, metastatic lymph node, portal vein tumor thrombus (PVTT) and non-tumor liver tissue. After aggregation of the primary tumor data of the 10 patients (Du, et al. 2021), R package “Seurat” was used for cell filtration, normalization, then we found the top-2000 highly variable genes for dimensional reduction and 6 clusters: carcinoma-associated fibroblasts (CAFs), endothelial cells (ECs), bursa dependent lymphocyte (B cells), macrophages, thymus-dependent lymphocyte (T cells) and tumors.

Moreover, we classified the cluster of tumors into three groups: 3_Positive LCSCs, 3_Negative liver cancer cells, and other cells. The Seurat functions “DotPlot” were applicated to show the expression of ten hub m^6^A regulatory factors in 3_Positive LCSCs and 3_Negative liver cancer cells.

### Human primary HCC cells, LCSCs and HCC cell lines

The methods of LCSCs isolation and culture were adopted in this study: tumor stem cells derived from liver cancer cell lines were cultured by low adsorption suspension into pellets, and Triple^+^ (CD133^+^CD24^+^EPCAM^+^) flow separation method (Zheng et al. 2018). Human primary tumor stem cells derived from clinical liver cancer tumor tissues were cultured on trophoblast by our research group (Park et al. 2015).

The primary HCC and LCSCs from HCC samples were gained from liver HCC patients who underwent surgery at Guangzhou First People’s Hospital. The method and human samples used in our study were approved by the ethics committee at Guangzhou First People’s Hospital of South China University of Technology and strict adherence to the Declaration of Helsinki. Separation of the primary HCC cells has been established as previously described (Xie et al. 2021), and the isolation of LCSCs was followed after the separation of HCC cells which were washed with Dulbecco’s modified Eagle medium Nutrient Mixture F-12 (HAM) for 3 times and resuspended in HAM added with 0.5% bovine serum albumin (BSA), 20 ng/mL Epidermal Growth Factor (EGF), 4 ng/mL Basic Fibroblast Growth Factor (bFGF) and 1% penicillin/streptomycin. We seeded these cells onto mouse embryonic fibroblasts (MEF) and incubated in a 5% CO2 and 37 °C incubator. The cells grew clonally on MEF and the medium was refreshed every day.

The cells of Hep3B and Huh7 were gained from ATCC and grew in a 5% CO2 and 37 °C incubator. The culture media were Dulbecco’s modified Eagle medium (DMEM) with 10% fetal bovine serum and 1% penicillin/streptomycin. Hep3B and Huh7 suspension cells (Hep3B SP and Huh7 SP) were cultured in HAM with 2% B27, 20 ng/ml EGF, 4 ng/ml bFGF, and 1% penicillin/streptomycin. Cells were cultured in a 5% CO2 and 37 °C incubator.

### Flow cytometry

Single-cell suspensions (Hep3B SP and Huh7 SP) were prepared and incubated with CD24 antibody (BioLegend Cat#311,106, RRID: AB_314855), EPCAM antibody (BioLegend Cat#324,212, RRID: AB_756086), and CD133 antibody (Biolegend, #S16016B, RRID: AB_2734479) for 30 min in 4 °C, followed by flow cytometry analysis. LCSCs (LCSC1 and LCSC2) were prepared and incubated with ALDH antibody (STEMCELL, Cat#01700) for 30 min in 37 °C, followed by flow cytometry analysis.

### RNA extraction and quantitative real-time PCR (RT-qPCR)

Total RNA was extracted using TRIzol reagent (TakaRa, Cat#9109) based on the manufacturer’s protocol. cDNA synthesis was operated using the PrimeScript™ Reverse Transcriptase kit (TakaRa, Cat#9767). The primers in Table S1 and Table S2 were used to perform qPCR.

### Consensus cluster analyses and PCA analyses

R package “ConsensusClusterPlus” and “PCA” was used to determine the function of ten hub m^6^A regulatory factors to divide HCC patients into various subgroups and study the gene expression patterns in the corresponding subgroups. After performing consensus clustering cumulative distribution function (CDF) for *k* = 2 to 9 in both TCGA and ICGC, *k* = 2 was shown and chosen for further study. To verify the significance of the cluster results of ten hub m^6^A regulators, we performed survival analysis and clinical information in TCGA and ICGC datasets.

### GSVA and functional annotation

R package “GSVA” was used to perform GSVA enrichment analysis, getting different biological processes between stratified subgroups. GSVA, using a non-parametric and unsupervised method, is a common way to estimate the variation in biological process activities and pathways in the samples of gene expression dataset. We obtained function sets of “c2.cp.kegg.v7.4.symbols.gmt” and “h.all.v7.4.symbols.gmt” from MSigDB dataset. Adjusted *p* < 0.05 and logFC > 0.1 was considered as statistically significant, and we got the top 100 different pathways between 2 models in KEGG and top 30 different pathways between 2 models in HALLMARK with “limma” *R* package.

### Establishment and verification of the prognostic model

UNICOX Analysis of ten candidate m^6^A regulatory factors (adjusted *p* < 0.05) was performed to confirm prognostic genes in ICGC. The LASSO analysis (adjusted *p* < 0.05) was applicated to construct a prognostic mode at the least and best risk factors. Then using the MULTICOX Analysis, we chose four regulators to build a prognostic risk signature. Finally, survival analysis, ROC analyses and risk score distribution in both TCGA and ICGC datasets were performed to verify the prognostic model.

### Establishment of a novel nomogram for individuals

We performed nomogram analyses with *R* “rms” to combinate this prognostic signature with relevant clinical information.

## Results

### Landscape of genetic variations in m^6^A regulatory factors related to stemness of HCC

**Fig. 1:**
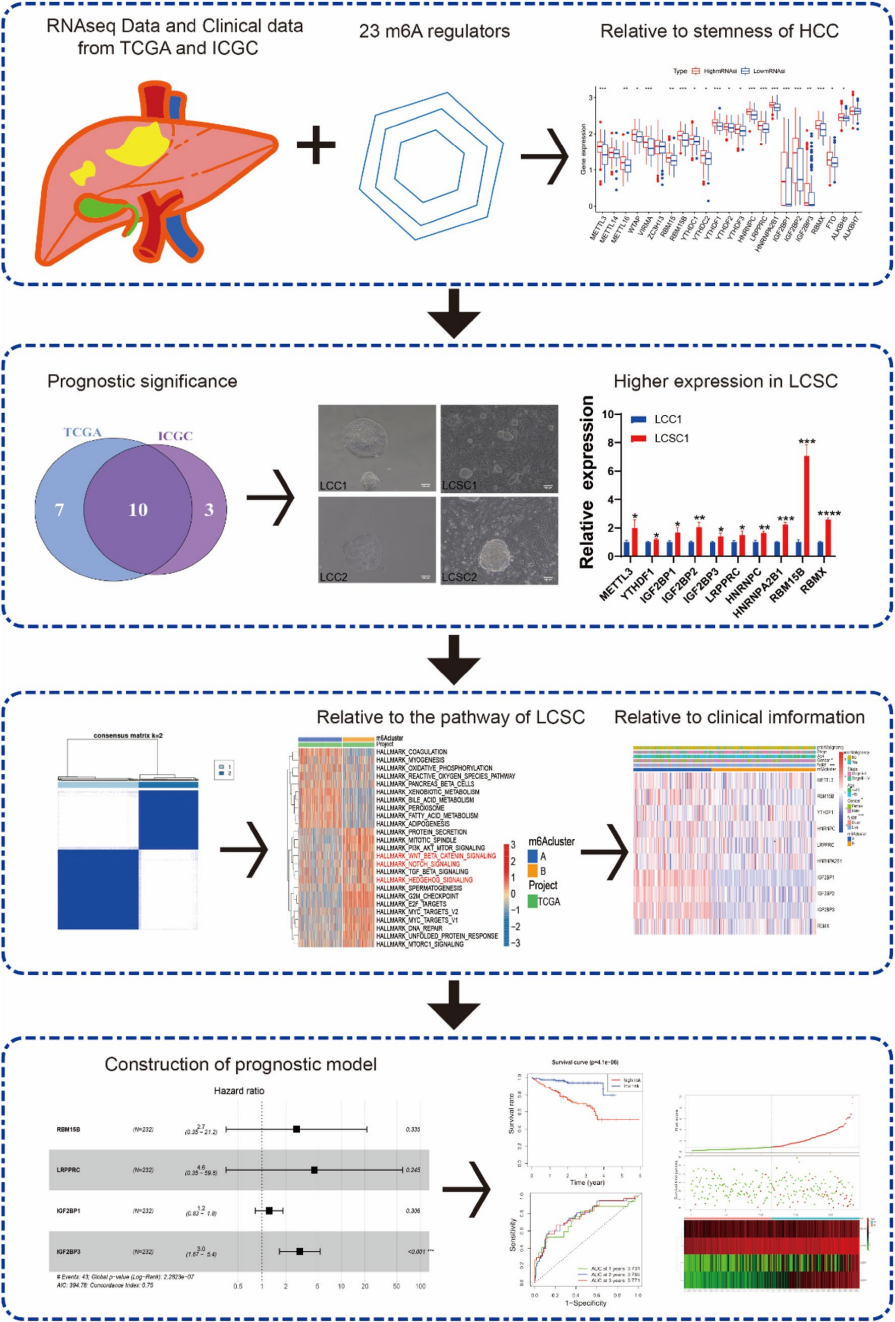
The whole flowchart was shown to reveal the analyses process of prognostic model of HCC and underlying associations with LCSCs based on ICGC-LIRI-JP and TCGA-LIHC databases

**Fig. 2:**
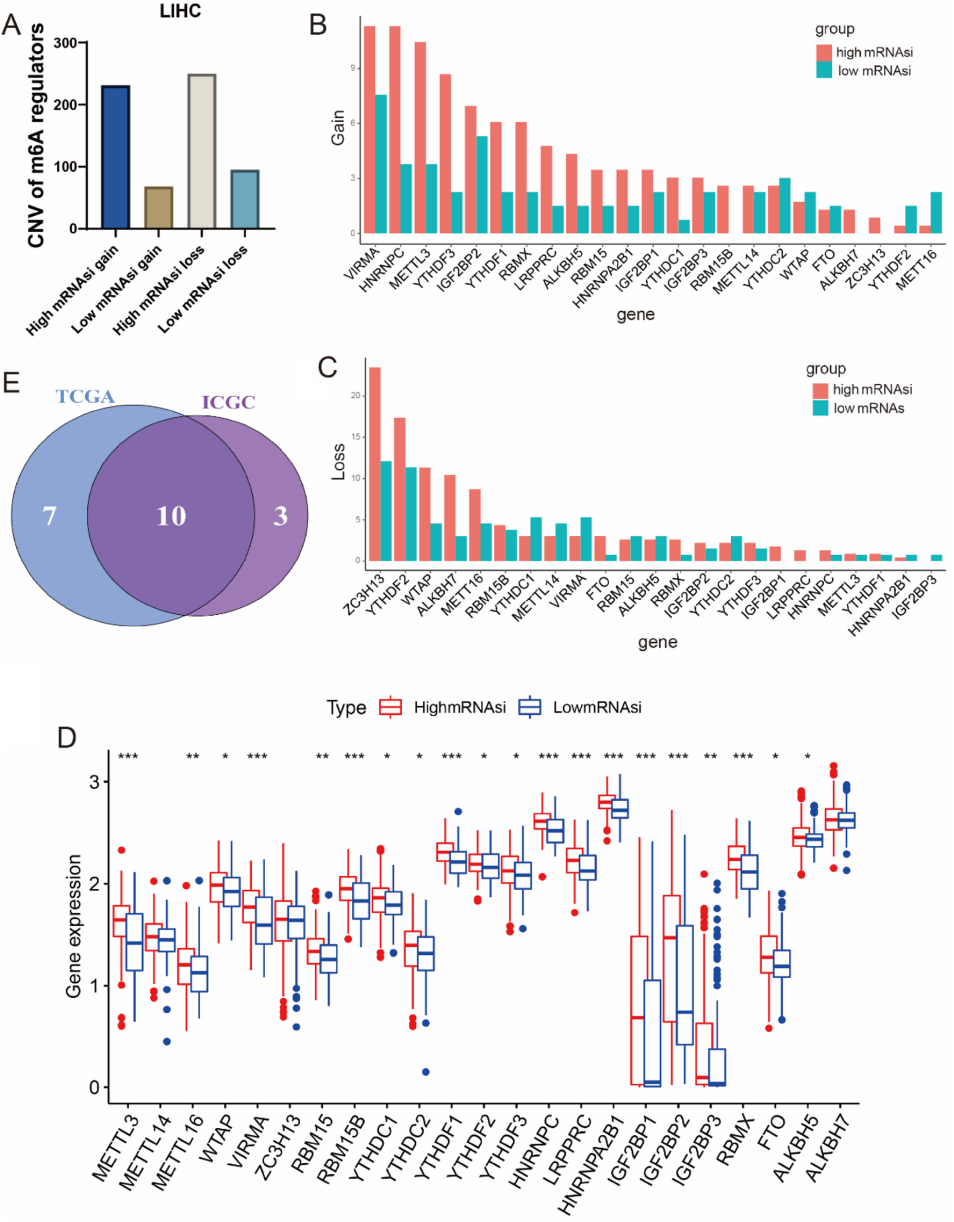
Expression and genetic landscapes of m^6^A DEGs in HCC patients with high- or low-mRNAsi in TCGA. **A** CNV frequency of in high- and low-mRNAsi in TCGA cohort. **B**, **C** Gain or loss copy number of 23 m^6^A regulatory factors in HCC patients with high- and low-mRNAsi in TCGA. **D** Expression level of 23 m^6^A regulatory factors in high- or low-mRNAsi in TCGA. **E** The overlapped prognostic m^6^A regulatory factors in TCGA and ICGC cohorts. Ten hub m^6^A regulatory factors were gained for detail analysis

The specific flowchart of our study is shown in Fig. 1. 23 m^6^A regulatory factors were finally identified in our work. mRNAsi index was utilized to analyze liver hepatocellular carcinoma (LIHC) cases in TCGA, which was selected to quantify the stem-like indices and well-calculated stemness index for HCC patients. We divided the LIHC patients into high-mRNAsi and low-mRNAsi group based on the median data of mRNAsi index. It has been confirmed that the expression level of m^6^A regulatory factors is correlated with CNV, and the change of CNV may be one of the main reasons causing the expression disorder of m^6^A regulatory factors (Li et al. 2021b; Li et al. 2019). In this study, we found that the CNV of 23 m^6^A regulatory factors showed a prevalent alteration and the CNV alteration frequency in the high-mRNAsi patients were higher than those in the low-mRNAsi patients (Fig. 2A). As the study showing that the CNV was correlated with stemness of HCC, most of m^6^A regulatory factors in high-mRNAsi patients tend to gain and loss copy number (Fig. 2B, C). Then, we compared the m^6^A regulatory DEGs between low- and high-mRNAsi patients. Interestingly, 23 m^6^A regulatory factors were remarkably highly expressed in high-mRNAsi patients among 371 samples (Fig. 2D). Excluding METTL14, ZC3H13 and ALKBH7, other 20 m^6^A regulatory factors were regarded as significance (adjusted *p* < 0.05) (Fig. 2D). We concluded that m^6^A regulatory factors expression was correlated with the stemness of HCC tissues, and the expression lever of m^6^A regulatory factors was higher in HCC tissues with high stemness.

### Survival analysis of m^6^A regulatory factors in TCGA and ICGC

To investigate the correlation between m^6^A DEGs which was related to stemness and prognosis of HCC patients, we found out 17 m^6^A DEGs in TCGA and 13 m^6^A DEGs in ICGC were considered significant (adjusted *p* < 0.05) by overall survival (OS) analysis in 20 m^6^A regulatory factors. After determining the shared regulatory factors in both TCGA and ICGC, ten hub regulatory factors were selected (Fig. 2E). The survival analysis of ten hub regulatory factors, including YTHDF1, IGF2BP1, METTL3, IGF2BP3, HNRNPA2B1, IGF2BP2, RBM15B, HNRNPC, RBMX and LRPPR were presented (Figure S1, S2). Furthermore, we found that higher expression of ten hub regulatory factors were correlated with poor prognosis for HCC patients, consistent with the tumorigenicity of m^6^A regulatory factors in the development of malignant tumors (Figure S1, S2).

### m^6^A regulatory factors were highly expressed in LCSC

**Fig. 3:**
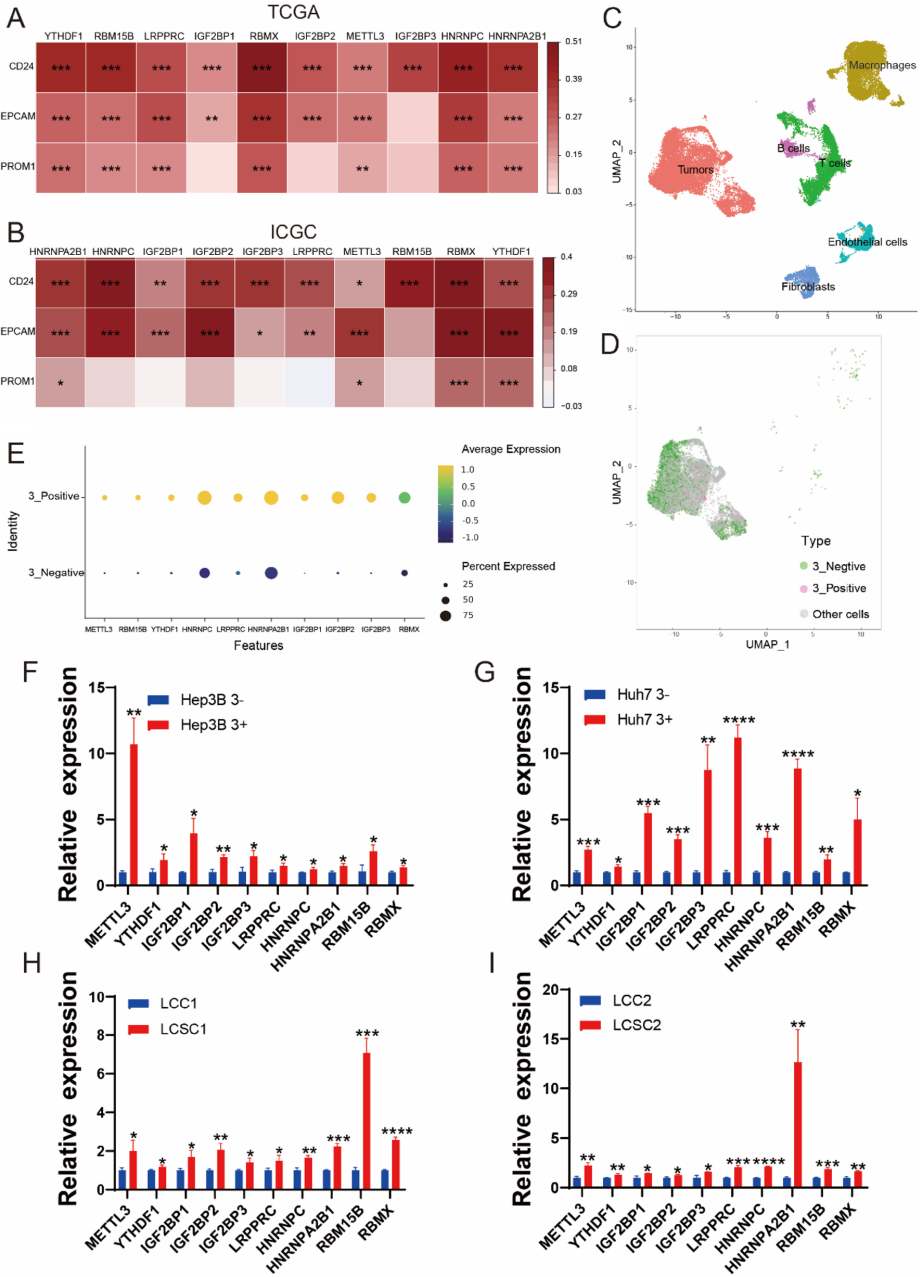
Stemness analyses of ten hub m^6^A regulators in TCGA, ICGC and scRNA-seq datasets. **A**, **B** Correlation analysis of ten hub m^6^A regulatory factors and three CSC markers in TCGA and ICGC database. *P < 0.05, **P < 0.01, ***P < 0.001. **C** UMAP plot of different cell types, pink represented tumor cells. **D** UMAP plot of 3_Positive (CD24^+^EPCAM^+^CD133^+^) LCSCs, 3_ Negative (CD24^−^EPCAM^−^CD133^−^) liver cancer cells and other cells in cluster of tumor cells. **E** Expression levels of ten m^6^A regulatory factors in 3_Positive (CD24^+^EPCAM^+^CD133^+^) LCSCs and 3_Negative (CD24^−^EPCAM^−^CD133^−^) liver cancer cells in dot diagram. **F**, **G** RT-qPCR analysis of ten m^6^A regulatory factors in 3_Negative (CD24^−^EPCAM^−^CD133^−^) liver cancer cells (Hep3B 3-, Huh7 3-) and 3_Positive (CD24^+^EPCAM^+^CD133^+^) LCSCs (Hep3B 3 + , Huh7 3 +). **H**, **I** RT-qPCR analysis for ten m.^6^A regulatory factors in primary liver cancer cells (LCC1, LCC2) and LCSCs (LCSC1, LCSC2)

Based on association between the expression and stemness of HCC, it promoted us to consider the relationship between m^6^A regulatory factors and the LCSCs which is known as tumor-initiating cells and is a small subpopulation of malignant tumor cells with stemness characteristics (Ahmed et al. 2017; Makena et al. 2020). Moreover, CSCs maintain the self-renewal ability by the assistance of m^6^A demethylation actively (Ma and Ji 2020). We analyzed the association between ten hub regulatory factors and three markers of CSCs. As a result, most of ten hub regulatory factors were positively related to CD24, EPCAM, and PROM1 (CD133), respectively, either in TCGA or ICGC patients (Fig. 3A, B). Then, we obtained a dataset (GSE149614), containing ten HCC samples from four relevant sites including: primary tumors, metastatic lymph nodes, portal vein tumor thrombus (PVTT) and non-tumor livers. After normalization, we collected ten HCC tumor samples in the dataset. Undergoing cell filtration, dimensional reduction, and clustering analysis, we found six clusters including: carcinoma-associated fibroblasts (CAFs), endothelial cells (ECs), macrophages, thymus bursa dependent lymphocytes (B cells), dependent lymphocytes (T cells), and tumors by markers of differential cells (Fig. 3C, Figure S3). Afterwards we calculated and selected the components of tumors for further analysis. CD24, EPCAM, and CD133, the markers of cancer stem cells (CSCs) reflected the stemness. Provided that the scRNA-seq data, we proposed to study the relationship between these ten hub m^6^A regulatory factors and these three markers of CSCs. Thus, we clustered the cell populations into three groups with 3_Positive (CD24^+^EPCAM^+^CD133^+^) LCSCs, 3_Negative (CD24^−^EPCAM^−^CD133^−^) liver cancer cells and other cells, respectively (Fig. 3D, Figure S4A–S4C).

Then, we discovered that m^6^A regulatory factors were almost highly expressed in the tumors of patients with 3_Positive LCSCs (Fig. 3E), this triggered us to consider that the high-expression m^6^A regulators may heighten the stemness of HCC cells and then led to poorer prognosis. In order to confirm our speculation, we sorted out 3_Positive (CD24^+^EPCAM^+^CD133^+^) LCSCs and 3_Negative (CD24^−^EPCAM^−^CD133^−^) liver cancer cells by flow cytometry in both Hep3B and Huh7 cells (Figure S4D, S4F, S4G), then we discovered that the expression of these ten m^6^A regulatory factors were highly expressed in 3_Positive (CD24^+^EPCAM^+^CD133^+^) LCSCs from Hep3B and Huh7 cells by RT-qPCR (Fig. 3F–G). For further verification, we isolated the LCSCs from human samples such as LCSC1 and LCSC2 (Figure S4E) which expressed stemness characteristics. Their expression of stemness marker ALDH was more than 70% and the expression ratios of stemness genes and stemness markers were the same or even higher than those of Hep3B SP and Huh7 SP cells (Figure S5A−E). Employing RT-qPCR analysis, we found that these ten hub regulatory factors were higher expressed in LCSCs (LCSC1 and LCSC2) than liver cancers (LCC1 and LCC2) (Fig. 3H, I). These results showed that these ten hub regulatory factors might be the significant factors to influence the stemness of HCC.

### HCC patients were divided into different subgroups by ten hub m^6^A regulatory factors

**Fig. 4:**
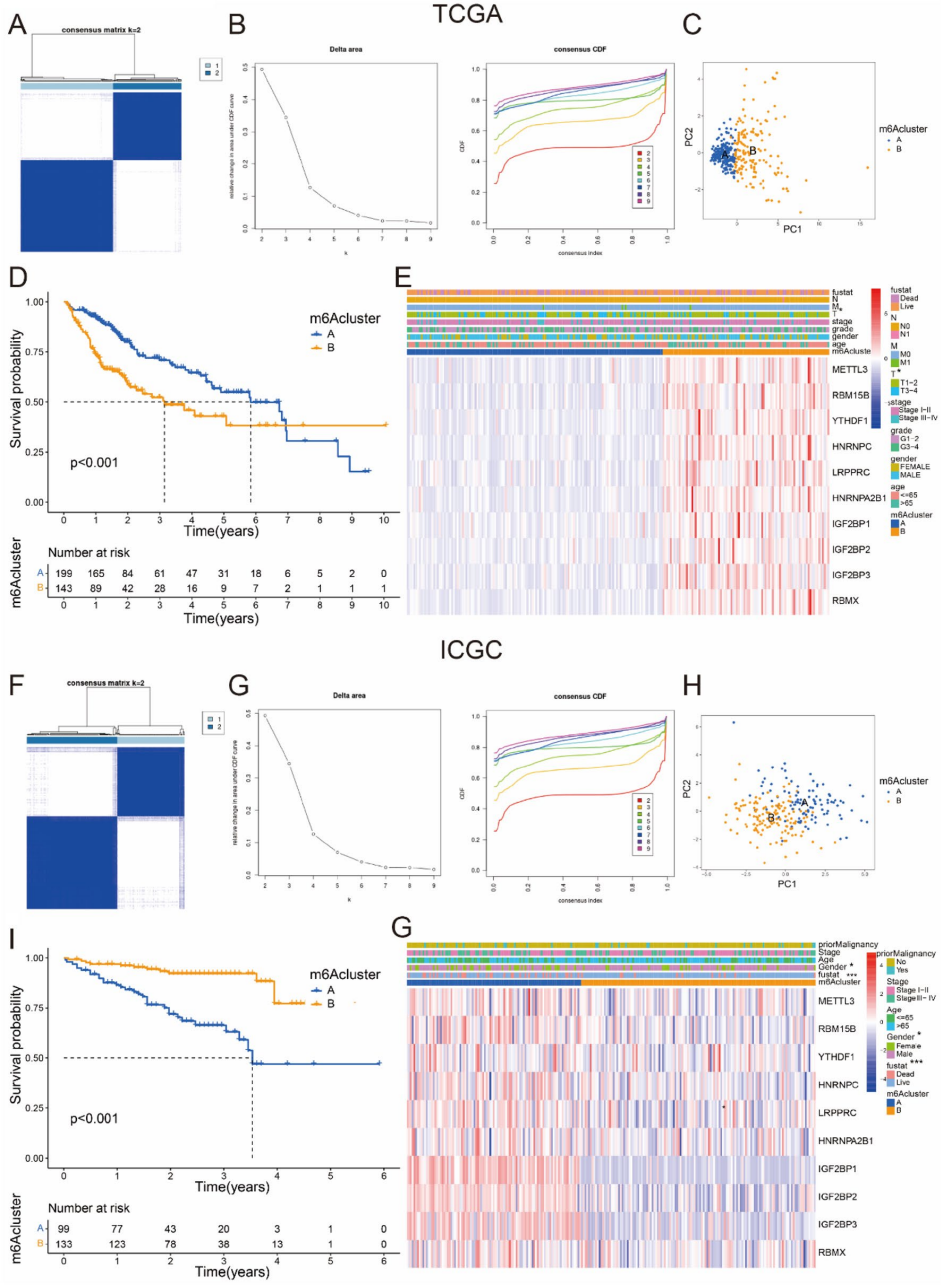
Consensus cluster and PCA by m^6^A regulatory factors. **A**, **B** CDF for k = 2 was shown and chosen for further study from *k* = 2 to 9 in TCGA. **C** PCA analyses of the gene expression matrix of patients with HCC in TCGA. **D** Survival analyses for HCC patients in TCGA. **E** Heatmap with clinicopathologic characteristics of the two subgroups (subgroup A and subgroup B) divided by consensus cluster of ten hub m^6^A regulators in TCGA. **F**, **G** CDF for *k* = 2 to 9 in ICGC, *k* = 2 was showed and chose for further study. **H** PCA analyses of gene expression matrix of HCC patients in ICGC. **I** Survival analyses for HCC patients in ICGC. **G** Heatmap of clinicopathologic characteristics of the two subgroups (subgroup A and subgroup B) defined by consensus expression of ten hub m^6^A regulatory factors in ICGC

Based on the important association between these ten hub regulatory factors with stemness characteristics, we classified HCC patients with qualitatively different molecular subgroups accorded to the gene expression of 10 hub regulatory factors and 2 distinct molecular subgroups were eventually identified by unsupervised clustering, which included 213 patients in subgroup A and 158 patients in subgroup B in TCGA dataset (Fig. 4A, B), while 104 patients in subgroup A and 139 patients in subgroup B in ICGC dataset (Fig. 4F, G). The results from ICGC dataset confirmed the correctness those from TCGA dataset. Then, we adopted PCA analyses to better visualize the subgroups, including TCGA-A, TCGA-B, ICGC-A, and ICGC-B. The whole gene expression matrix of patients with HCC could be categorized into two distinct groups by PCA analyses, confirming the robustness of the existence of these two subgroups in both TCGA and ICGC datasets (Fig. 4C, H). In addition, we found that the subgroups separated by these ten hub m^6^A regulators had a marked different prognosis of patients with HCC. In TCGA dataset, TCGA-B subgroup in which the expression of ten hub regulatory factors was higher had a worse prognosis than TCGA-A subgroup did (Fig. 4D, E). As well in ICGC dataset, the ICGC-A had worse prognosis when compared with ICGC-B that was correlated with higher expression of ten hub regulatory factors (Fig. 4I, G). Furthermore, we studied the relationship of clinicopathological characteristics and different subgroups in ICGC and TCGA. The TCGA-B with higher expression level of m^6^A regulatory factors was at higher stage of *T* (adjusted *p* < 0.05) (Fig. 4E), while the ICGC-A subgroup with higher gene expression was significantly associated with gender (adjusted *p* < 0.05), and with worse survival status (adjusted *p* < 0.001) (Fig. 4G). We concluded that m^6^A regulators expression data in ICGC were more correlated with the survival and prognosis of HCC patients.

### Function characteristics in different subgroups

**Fig. 5:**
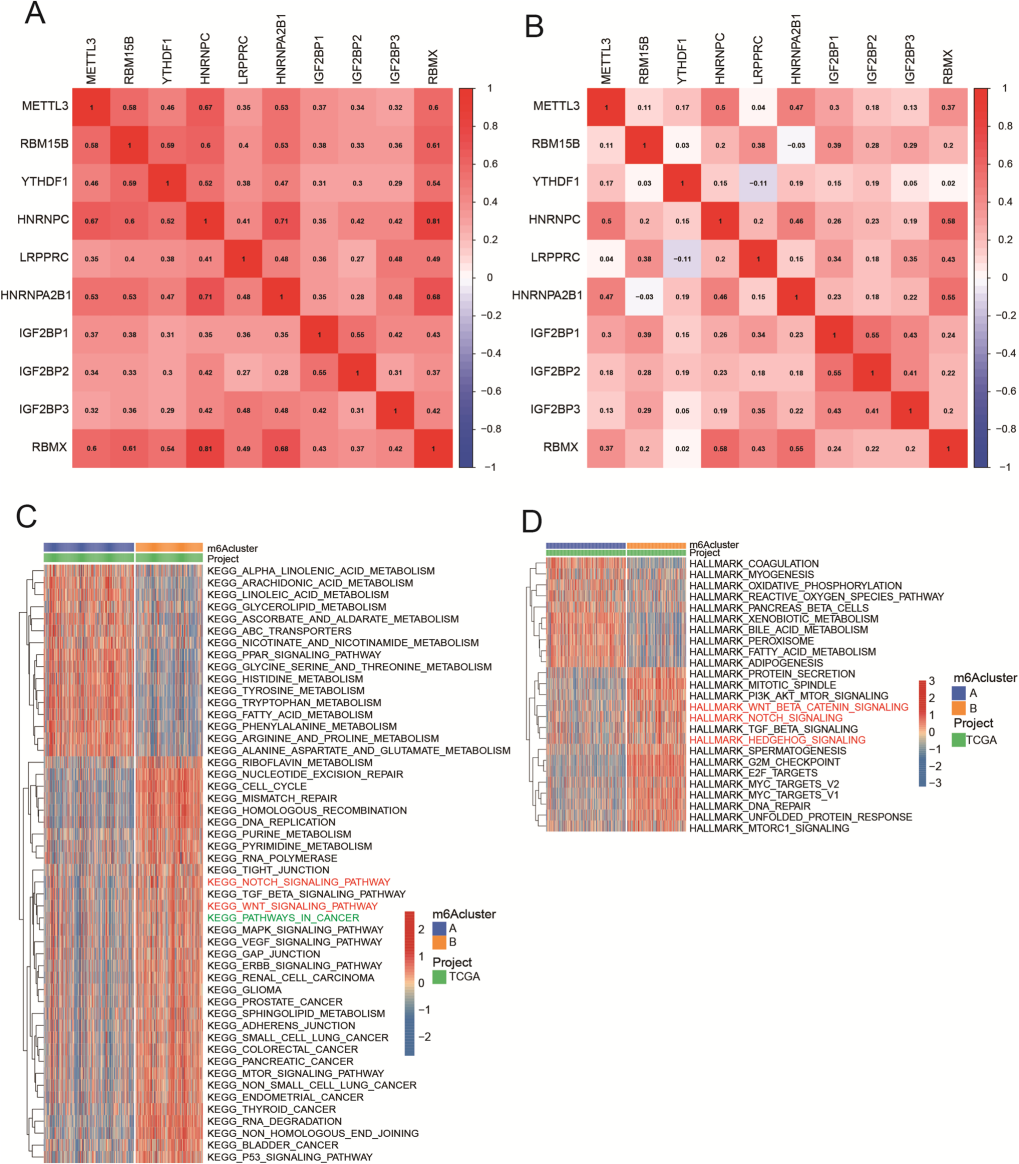
Correlation and function analysis of prognostic m^6^A DEGs in TCGA and ICGC. **A**, **B** The associations of ten overlapped DEGs with prognostic relevance in TCGA and ICGC. The closer relationship between the overlapped regulatory factors, the closer correlation value is to 1, the redder the square. **C**, **D** The GSVA of ten hub regulatory factors based on KEGG and HALLMARK annotation in TCGA. The functionality of the red and green logo was what we focus on

To better study the function of ten hub m^6^A regulatory factors, correlation analysis was conducted that ten hub regulatory factors exhibited strong correlation with each other with strict criteria (adjusted *p* < 0.05) in TCGA and ICGC (Fig. 5A, B). In addition, the relativity of hub regulatory factors in TCGA was more close than those in ICGC, thus TCGA database was used as the main database for functional analysis. On the other hand, to determine how the molecular subtypes affected the prognosis, we performed GSVA of the two subgroups accorded to gene expression of ten hub m^6^A regulatory factors in KEGG and HALLMARK annotation. As shown in Fig. 5C, compared with the cluster A, clusters B which highly expressed ten hub regulatory factors was active in methylation processes, cell cycle and tumor malignancy. In contrast, cluster A was enriched in a variety of metabolic processes. Giving various pathways in the KEGG annotation were enriched such as “bladder cancer”, “thyroid cancer”, “colorectal cancer”, “glioma”, “renal cell carcinoma”, and “pathways in cancer” (Fig. 5C). Therefore, we concluded that ten hub regulatory factors played critical role in a variety of cancers and may be promising therapeutic targets.

Furthermore, as we had showed the expression of these ten hub regulatory factors was more highly expressed in high-mRNAsi HCC patients, indicating the potential function of carcinogenesis was correlated with CSCs. And another result supported this conclusion that to show that the developmental signaling pathways including Notch signing pathway, Hedgehog signaling, and Wnt beta catenin signaling were ranked as the top (Fig. 5C), which sustained CSC alive (Clara et al. 2020). Consistently, HALLMARK annotation for the m^6^A DEGs among clusters A and B, the Notch, Hedgehog, and Wnt beta catenin signaling pathway also ranked as the top and is shown in Fig. 5D. These function results showed that these ten hub regulatory factors might affect the survival and development of CSCs in HCC patients.

### A prognostic model was established in ICGC dataset and verified in TCGA dataset

**Fig. 6:**
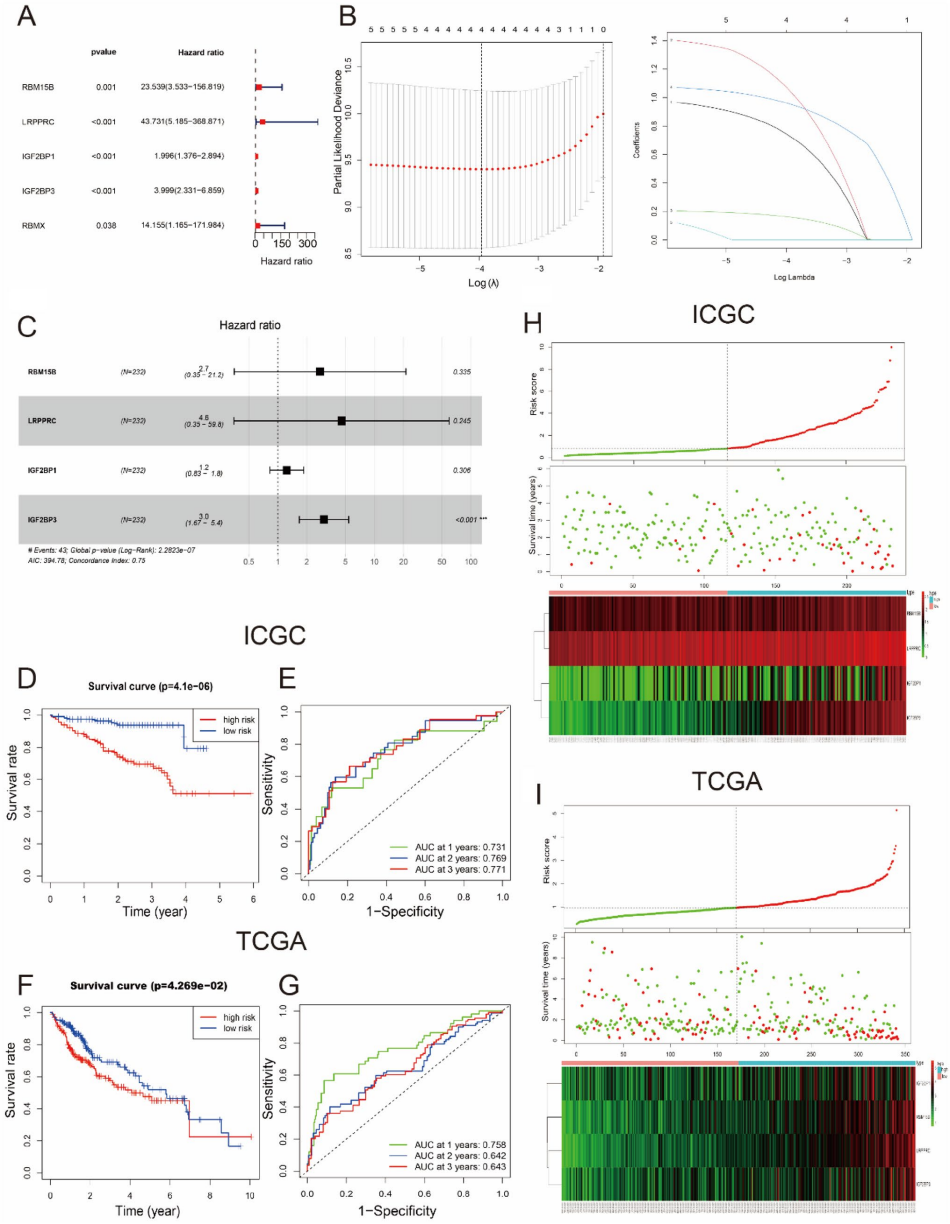
Construction and validation of the m^6^A regulatory factors model. **A** UNICOX analyses of ten m^6^A regulators for patients with HCC in ICGC which is as training dataset in this study (adjusted *p* < 0.05). **B** Establishment of the LASSON regression model. **C** MULTICOX analyses of RBM15B, LRPPRC, IGF2BP1, and IGF2BP3 in ICGC and the prognostic model was established with concordance index being 0.75. As following was the formula: RiskScore = 2.73 × RBM15B + 4.58 × LRPPRC + 1.23 × IGF2BP1 + 3.00 × IGF2BP3. **D**, **F** OS analyses of patients with HCC accorded to the RiskScore model in ICGC and in the verification dataset (TCGA). **E**, **G** The ROC analyses of ICGC and TCGA were calculated by RiskScore model at 1, 2, and 3 years. **H**, **I** Heatmaps and distribution of four regulatory factors in the high- and low-risk subgroups in ICGC and TCGA datasets, respectively. The green curve was low-risk and the red curve was high-risk. The green circle means alive and the red circle means dead. The RiskScore of patients with HCC in verification dataset was gained by risk score model and split in two groups based on the middle score of the RiskScore in training dataset

Giving the close relationship between ten hub regulators and prognosis of HCC, we adopted a UNICOX analyses based on the expression date of these ten m^6^A regulatory DEGs in ICGC to study its prognostic value (Fig. 6A). The results indicated that five out of ten hub regulatory factors were closely correlated with clinical outcomes (adjusted *p* < 0.05). These five regulatory factors, including RBM15B, LRPPRC, IGF2BP1, RBMX, and IGF2BP3, were all oncogenes with hazard ratio (HR) > 1(Fig. 6A). Then, we performed LASSON analyses to better identify the hub m^6^A regulatory factors with the strongest prognostic power. Consequently, only four regulatory factors were selected to build the prognostic model with corresponding coefficients from the LASSO algorithm (Fig. 6B). In addition, we utilized the MULTICOX analysis to confirm the consequence and gain the corresponding coefficients, and the specific formula to calculate the RiskScore was as follows: RiskScore = 2.73 × RBM15B + 4.58 × LRPPRC + 1.23 × IGF2BP1 + 3.00 × IGF2BP3 (Fig. 6C). To explore the prognostic function of this four-regulator prognostic model, we calculate the RiskScore based on this risk score model and, respectively, separated HCC patients into low-risk and high-risk clusters accorded to the median RiskScore in both the discovery cohort (ICGC) and the validation cohort (TCGA). As the OS curve analyses had demonstrated, high-risk HCC patients had worse prognosis compared with HCC patients with low-risk (adjusted p < 0.001) (Fig. 6D, F). Then, we adopted a ROC curve analyses and evaluated the area under ROC curve (AUC). The AUC of the discovery cohort in ICGC were 0.731, 0.769, and 0.771 for the 1-, 2-, and 3-year OS, while the AUC in TCGA were 0.758, 0.642, and 0.643 for the 1-, 2-, and 3-year OS, indicating great predictive ability for survival status (Fig. 6E, G). Ultimately, the risk score distribution of HCC patients, related survival status and heatmap displayed the expression of four m^6^A prognostic regulatory factors in both high-risk and low-risk was exhibited in Fig. 6H, I). As a result, we discovered that the higher the RiskScore was, the higher prognostic regulators’ expression, the worse the survival outcome.

### Construction of a prognostic nomogram for HCC patients

**Fig. 7:**
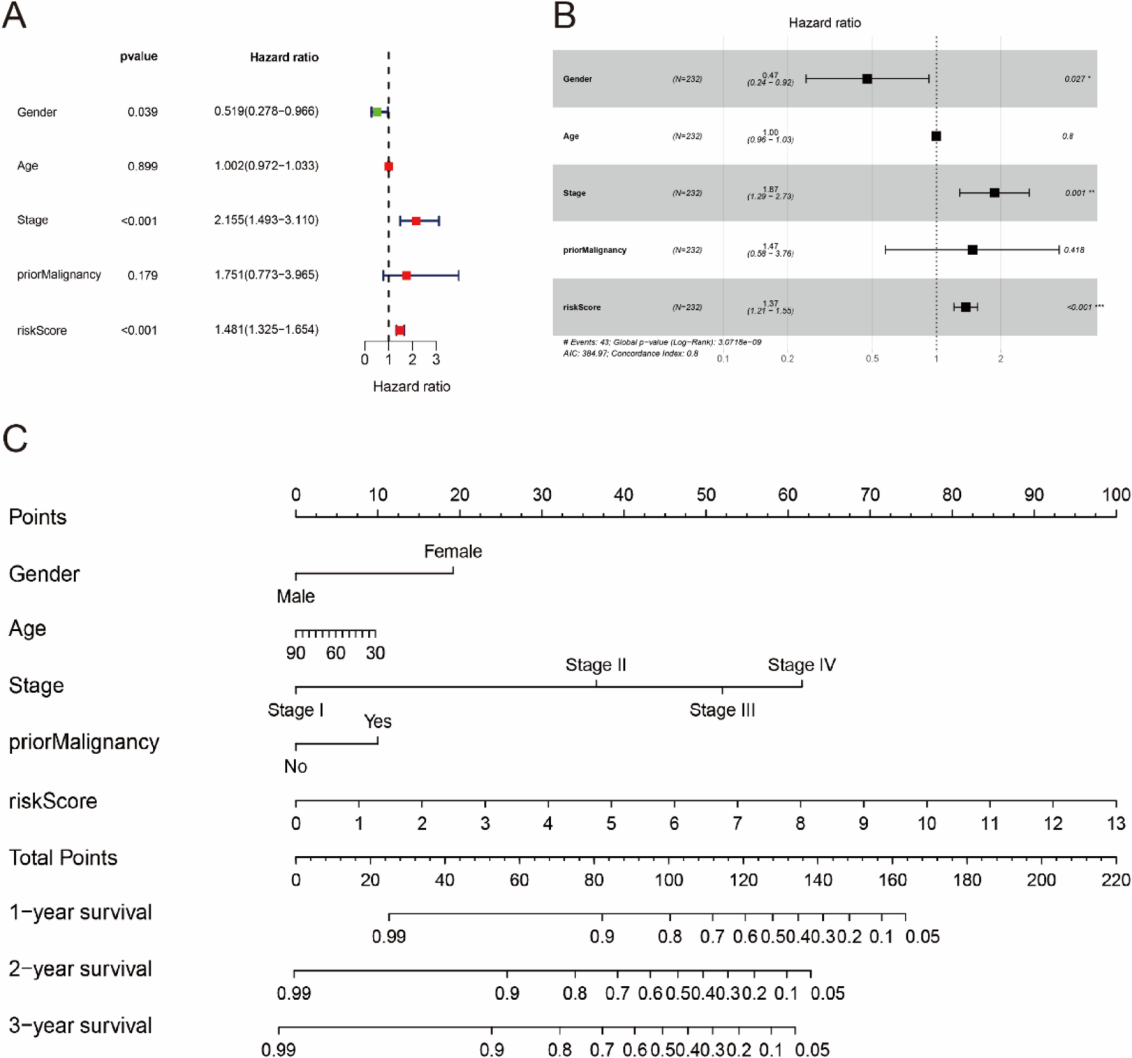
An innovative nomogram for ICGC risk score model. **A** UNICOX analyses of clinical characteristics. **B** MULTICOX analyses of clinical characteristics. **C** A nomogram according to RiskScore model and clinical-related characteristics

Giving that a synthetic analysis could better predict the clinical prognosis for HCC patients, UNICOX and MULTICOX analysis were performed to figure out the association between the HCC patients and clinically relevant information. The result showed that RiskScore, the stages of disease progression, and the gender, were the critical risk factors of HCC patients (adjusted *p* < 0.05) (Fig. 7A, B). We made a nomogram based on gender, age, prior malignancy, stage, and RiskScore to provide a quantitative method to forecast the clinical outcome of individuals (Fig. 7C). As shown in Fig. 7C, the novel nomogram could comprehensively forecast the 1-, 2-, and 3-year survival outcome of HCC patients. Furthermore, we found that the RiskScore was the most significant factor of all clinical variables.

## Discussion

Hepatocellular carcinoma (HCC) ranking third in cancer deaths worldwide is a serious disease (Siegel et al. 2021; Sung et al. 2021). The recurrence rate of HCC remains high, although many patients can be early diagnosis and treatment (Siegel et al. 2021). For patients whose disease has entered the middle and late stages, the prognosis is much less optimistic. Moreover, CSCs are known to work in the development of cancer, relapse and resistance after treatment. The characterization of CSCs and its molecular basis may provide many benefits for cancer treatment and therapy. Furthermore, m^6^A RNA modifications can be modified by “writes” and removed by “erases” (Fu et al. 2014). Differential expression of specific m^6^A regulatory factors was correlated with abnormally regulated RNAs in cancers; however, the same m^6^A regulatory factors may play different roles in different cancers (Chen et al. 2019; Huang et al. 2020). It is also well known that m^6^A RNA modification has a crucial function in regulating the generation, maintenance, and drug resistance with CSCs (Cui et al. 2017). The biological basis and functional characteristics of m^6^A regulatory factors may be innovative indicators and targets.

Previous study has shown that m^6^A regulatory factors could exert effectively in various tumors and m^6^A methylation is highlighted in relation to CSCs and cancer biology. For example, Jeremy et al. found that m^6^A methylation was generally upregulated in glioblastoma and the “writers” YTHDF2 specifically stabilizes MYC mRNA in CSCs of glioblastoma (Dixit et al. 2021). Likewise, Zhang CZ et al. suggested that YTHDF2 regulated the expression of OCT4 through m^6^A methylation, promoting the phenotype LCSCs and tumor metastasis (Zhang et al. 2020). Studies have shown that FTO plays a key role in the self-renewal and immune evasion of CSCs and indicates the great prospects of cancer therapy by targeting FTO (Su et al. 2020). Giving the study of m^6^A in CSCs, we found that m^6^A mechanism might serve as effective strategies and new target for elimination of CSCs. In this study, we focused on the comprehensive analyses of TCGA and ICGC databases. The analyses of the m^6^A regulatory DEGs in high-mRNAsi and low-mRNAsi were conducted in TCGA, and relevant OS analyses of hub regulators were performed in ICGC and were almost confirmed in TCGA. The data of scRNA-seq with HCC were also performed to verify the positive association between m^6^A regulatory factors and LCSCs. Furthermore, besides the consensus analysis and nomogram, we also successfully build a prognostic model of m^6^A regulators for HCC which was established in ICGC verified in TCGA databases, offering a prospective m^6^A-related target for patients with HCC.

Ten overlapped m^6^A regulatory DEGs had been gained in both TCGA and ICGC in our study, these ten hub m^6^A regulatory factors were upregulated in high-mRNAsi groups and showed worse prognosis in HCC patients. As a result, we figured out that m^6^A regulatory factors might be a prospective target for CSCs in HCC, then by analyzing the scRNA-seq data of HCC and followed by RT-qPCR validation, we found that the ten hub m^6^A regulatory factors were positively related to the CSCs markers CD24, EPCAM, and CD133 and highly expressed in LCSCs. Besides the positive correlation with CSCs markers, the results of GSVA function analysis of KEGG and HALLMARK enrichment also showed that the enriched terms were not only related to cell cycle, immune modifications and tumor malignancy, but also to the developmental signaling pathways of CSCs. The results of function analysis showed that these ten hub regulatory factors might affect the survival and development of CSCs in HCC patients. Moreover, we used UNICOX, LASSO, and MULTICOX analysis to select four risk factors (RBM15B, LRPPRC, IGF2BP1 and IGF2BP3) for establishing prognostic model. RBM15B, RNA-binding protein, performs as a key regulator of m^6^A methylation of RNA. Jaffrey SR’s team demonstrated that RBM15 and related RBM15B interacted with WTAP to form the complex to target mRNA and also were critical for XIST-mediated gene silencing (Patil et al. 2016). Leucine-rich PPR motif protein (LRPPRC), a member of the PPR family, was a known genetic mutation. The team from Liu et al. found that SNHG17 could inhibit C-MYC ubiquitination by interacting with LRPPRC in HCC and promote HCC proliferation through snHG17-LRPPRC-C-MYC regulatory axis, which provides a potential target for cancer therapy (Liu et al. 2021). As the member of the insulin-like growth factor2 mRNA binding protein family, IGF2BP1 can promote the stability of MGAT5 mRNA by upregulating m^6^A modification of MGAT5, thus maintaining the LCSCs phenotype (Yang et al. 2021). IGF2BP3, the protein encoded by this gene was primarily found in the nucleolus, and also known as that HBV-pgRNA (pregenomic RNA) could be a potential biomarker to predict clinical outcome and recurrence of HCC (Ding et al. 2021), thus the pgRNA-IGF2BP3 axis had a crucial function in HBV-associated liver cancer. In addition, IFN-α-2A could augment the level of m^6^A RNA modification to reduce pgRNA stability, thereby controlling the occurrence of HBV-associated HCC. The built RiskScore with those four factors RBM15B, LRPPRC, IGF2BP1, and IGF2BP3 were likely to divide HCC patients into two prognosis-related clusters and further verified by survival, ROC, UNICOX and MULTICOX analysis in this study. In summary, we analyzed RNA-seq data of m^6^A regulatory factors employing ICGC and TCGA datasets. In addition, the RiskScore feature and prognostic graph were inferred to comprehensively forecast the clinical outcome for patients with HCC. These consequences suggest that ten hub regulators are promising targets for potential therapeutic strategies, and clinical and experimental validation are needed to further verify the clinical function and more potential applications of this model.

## Supplementary Information

Below is the link to the electronic supplementary material.

Supplementary file1 (DOCX 2239 KB)

## Data Availability

No data were used to support this study.

## Abbreviations

scRNA-seq: Single-cell RNA-sequencing
RNA-seq: RNA-sequencing
HCC: Hepatocellular carcinoma
CSCs: Cancer stem cells
m^6^A: N6-Methyladenosine
OS: Overall survival
GSVA: Gene set variation analysis
APC: Principal component analysis
UNICOX: Univariate cox regression
LASSON: Least absolute shrinkage and selection operator
MULTICOX: Multivariate cox regression
OCLR: Single-class logistic regression
mRNAsi index: The stemness index based on mRNA expression
TCGA: The cancer genome Atlas
ICGC: The International Cancer Genome Consortium
LCSCs: Liver cancer stem cells
CNV: Copy number variation
GEO: Gene Expression Omnibus
CAFs: Carcinoma-associated fibroblasts
ECs: Endothelial cells
B cells: Bursa-dependent lymphocyte
T cells: Thymus-dependent lymphocyte
HAM: Dulbecco’s modified Eagle medium Nutrient Mixture F-12
BSA: Bovine serum albumin
EGF: Epidermal growth factor
bFGF: Basic fibroblast growth factor
MEF: Mouse embryonic fibroblasts
DMEM: Dulbecco’s modified Eagle medium
CDF: Consensus clustering cumulative distribution function
LIHC: Liver hepatocellular carcinoma
AUC: Area under ROC curve
