# Supplementary material for: Establishment of a prognostic model based on m6A regulatory factors and stemness of hepatocellular carcinoma using RNA-seq data and scRNA-seq data
Source: J Cancer Res Clin Oncol. 2023 Jul 19;149(14):12881–96. doi: 10.1007/s00432-023-05045-x (PMC10587019; doi:10.1007/s00432-023-05045-x)
Supplement: Supplementary file 1 — Supplementary file1 (DOCX 2239 KB) [file 432_2023_5045_MOESM1_ESM.docx]

**Supplementary Figure 1:**

**Survival analyses of m^6^A regulatory DEGs in TCGA dataset.** 10 overlapped m^6^A regulatory factors were showed. Red curve means high-expression and blue curve means low-expression of m^6^A regulatory factor. “p < 0.05” was adjusted.

**Supplementary Figure 2:**

**Survival analysis of m^6^A regulatory DEGs in ICGC dataset.** 10 overlapped m^6^A regulatory factors were showed. Red curve means high-expression and blue curve means low-expression of m^6^A regulatory factor. “p < 0.05” was adjusted.

**Supplementary Figure 3:**

**The markers of different cells in scRNA-seq datasets.** UMAP plot of the CD2, CD3D, and CD3E which represented the cluster of T cells; UMAP plot of the CD79A, CD79B, and MS4A1 which represented the cluster of B cells; UMAP plot of the CDH5, KDR, and PECAM1 which represented the cluster of ECs; UMAP plot of the ACTA2 which represented the cluster of CAFs; UMAP plot of the CD14, CD68, and CD163 which represented the cluster of macrophages; UMAP plot of the AFP, EPCAM, ALB, KRT18, ASGR1, and GPC3 which represented the cluster of tumors.

**Supplementary Figure 4:**

**Cell morphology and flow cytometry of LCSCs**. (A-C) UMAP plot of the expression levels of the three CSC markers PROM1(CD133), CD24 and EPCAM respectively in liver cancer cells. (D) Morphologies of liver cancer cells (Hep3B and Huh7) and LCSCs (Hep3B SP and Huh7 SP). (E) Morphologies of primary liver cancer cells (LCC1 and LCC2) and LCSCs (LCSC1 and LCSC2). (F-G) Flow cytometry analysis for CD133, CD24, and EPCAM in Hep3B SP and Huh7 SP cells.

**Supplementary Figure 5:**

**Characterization of stemness in LCSCs.** (A, B) Flow cytometry analysis for ALDH in LCSC1 and LCSC2. (C) Expression of stemness markers in liver cancer cells (Hep3B) and LCSCs (Hep3B SP) by RT-qPCR. (D) Expression of stemness markers in primary liver cancer cells (LCC2) and primary LCSC (LCSC2) by RT-qPCR. (E) Flow cytometry analysis for CD133, CD24, EPCAM, and ALDH in Hep3B SP, Huh7 SP cells, and LCSC2 respectively.


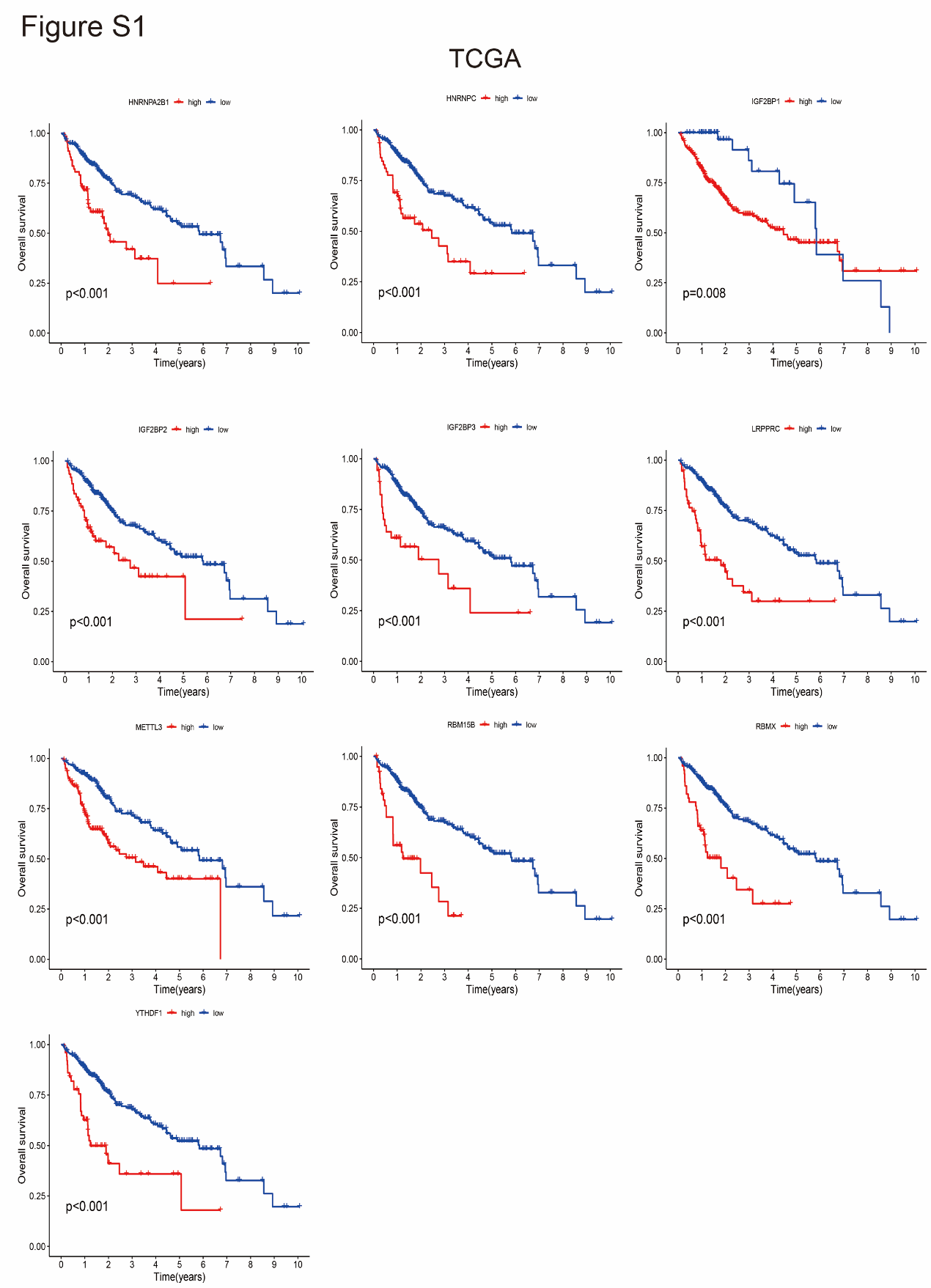

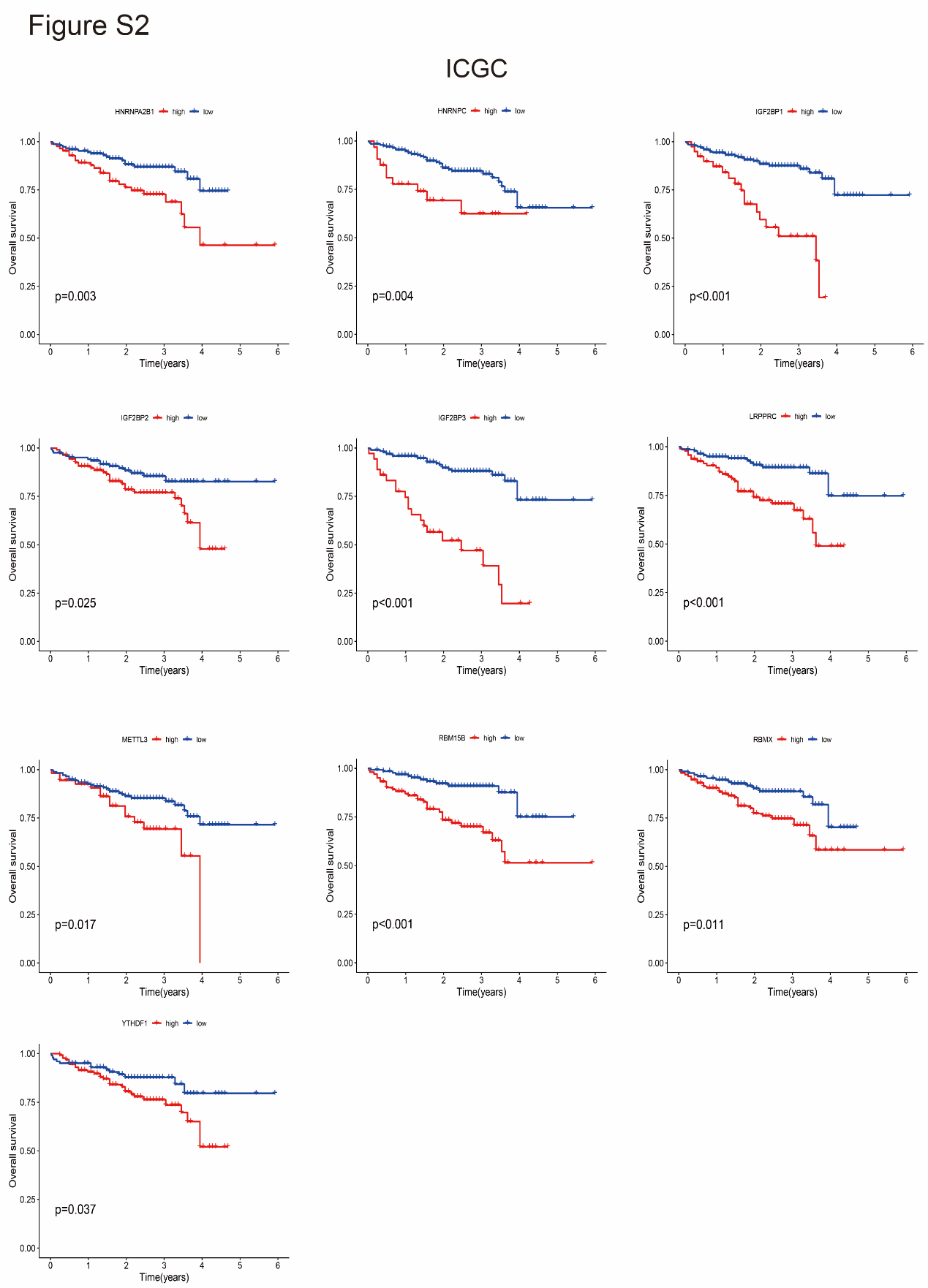

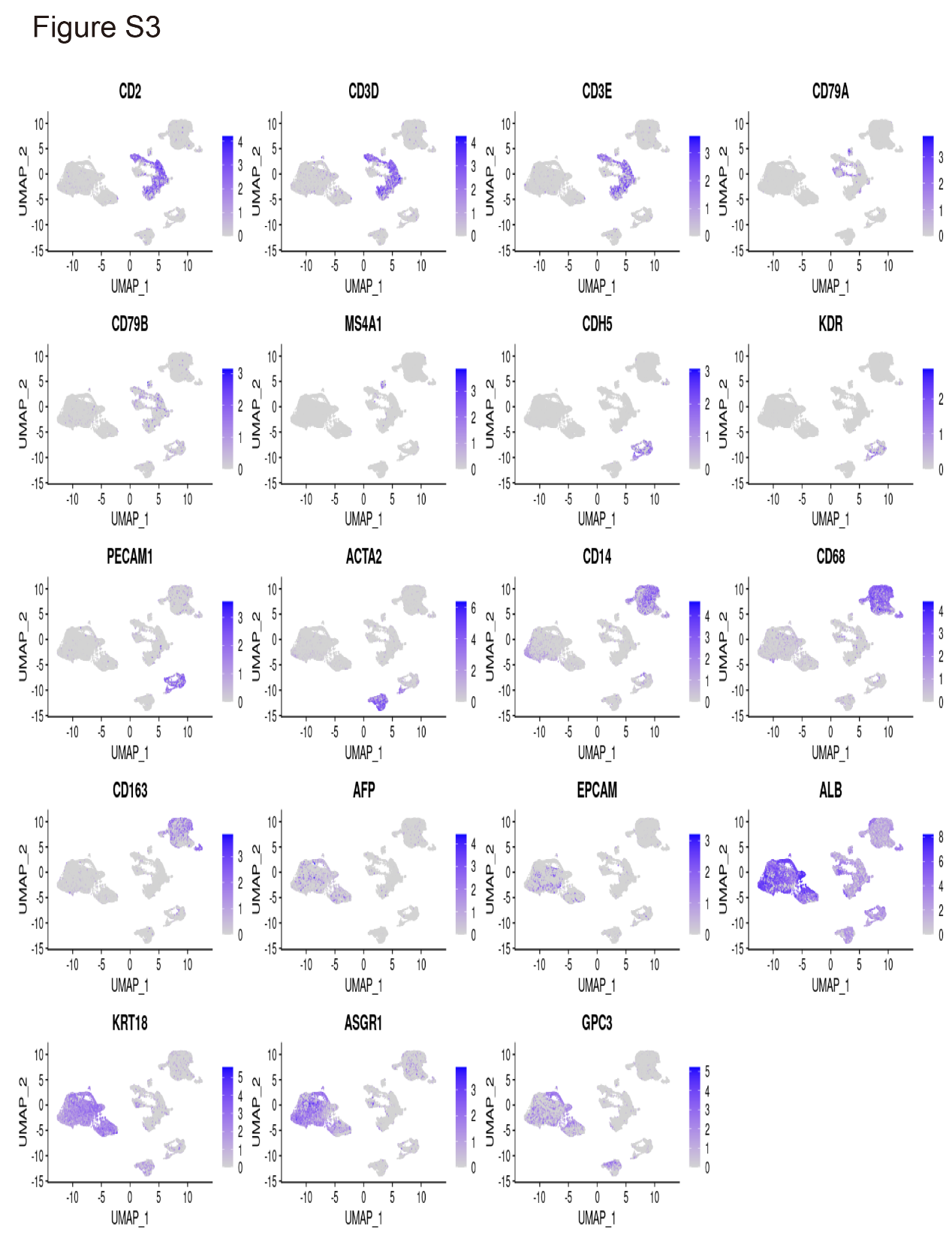

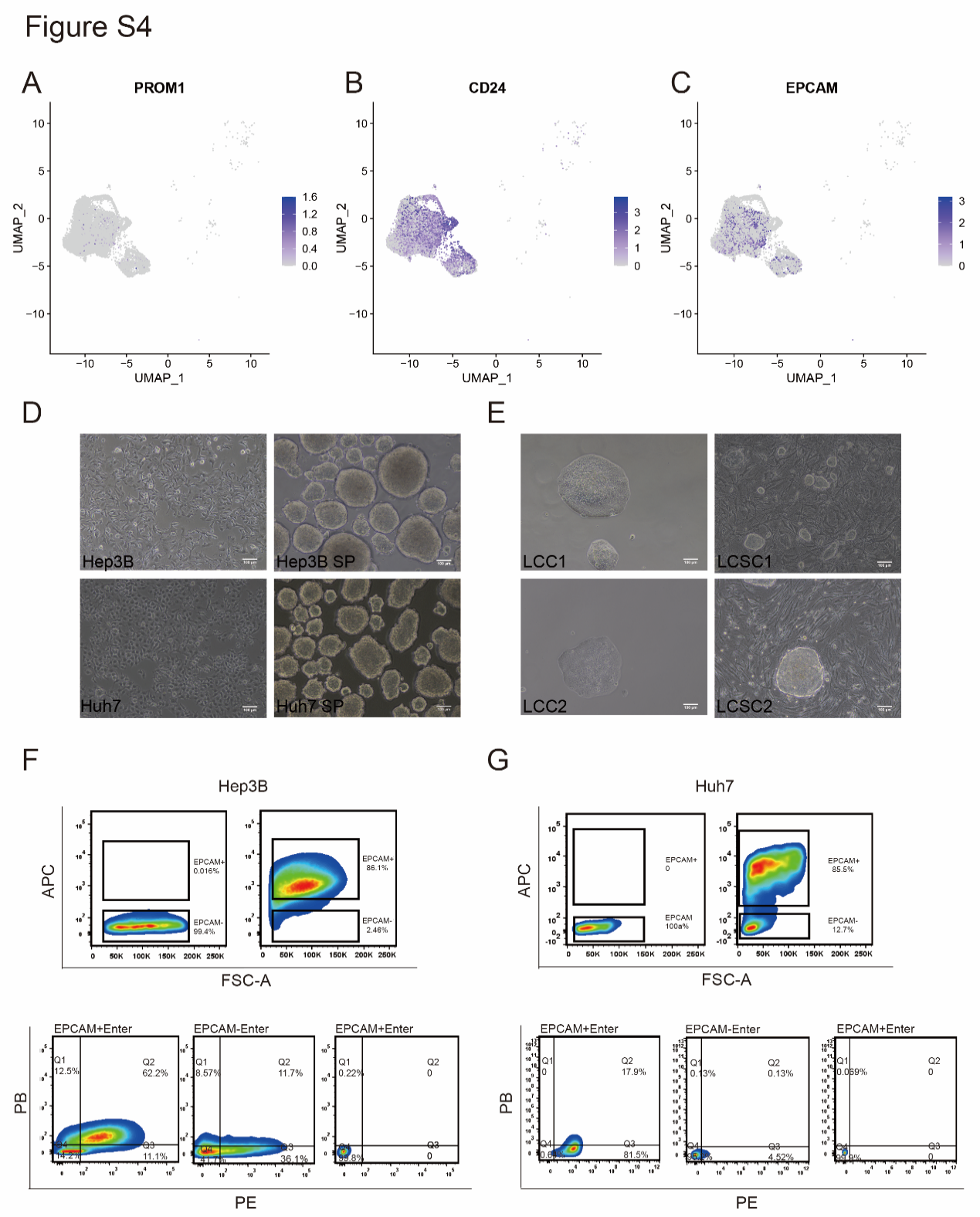

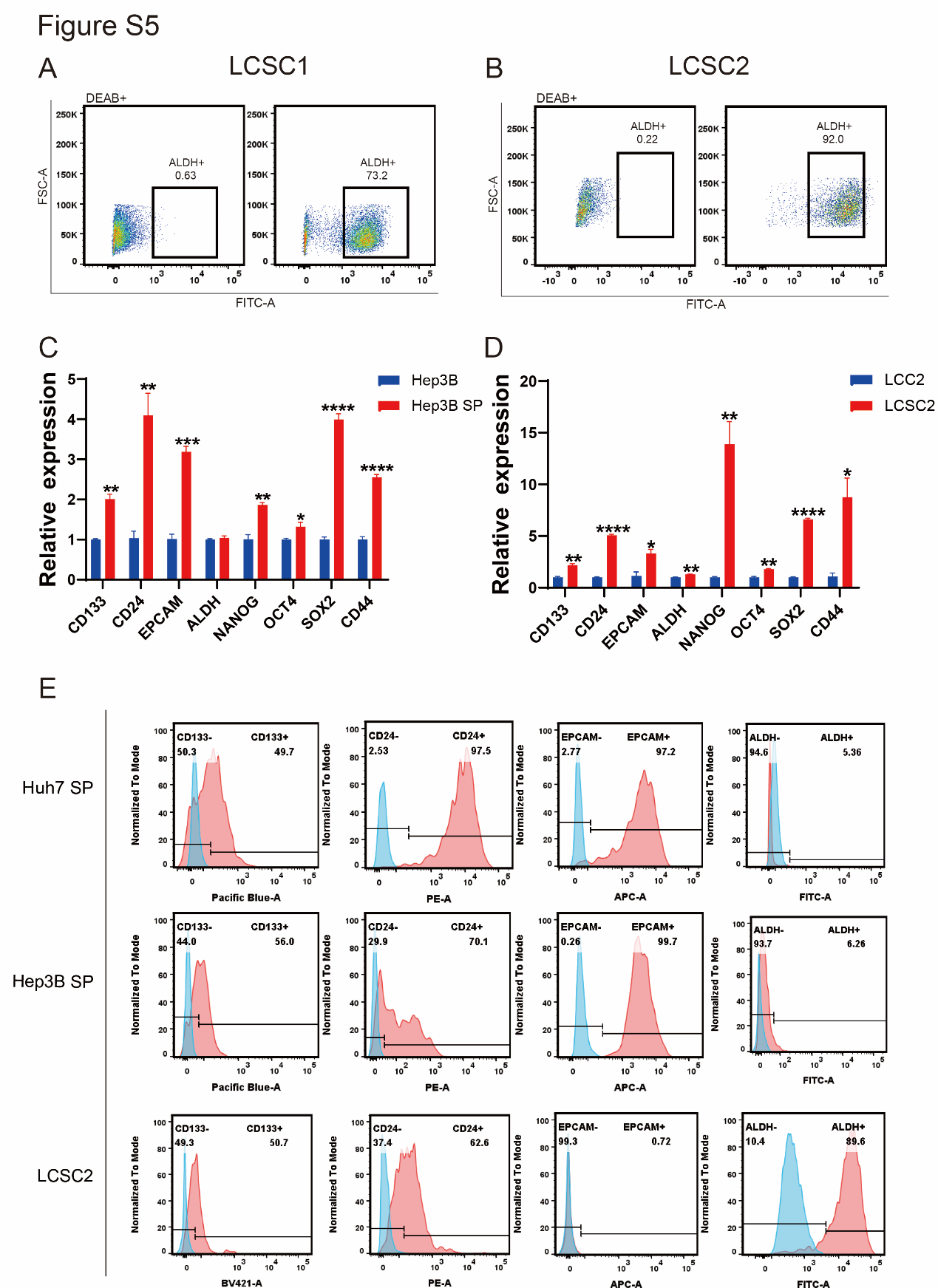


**Supplementary Table 1:**

**Primers used in RT-qPCR.**

| **Genes** | **Primers** |
| --- | --- |
| YTHDF1 | CAAGCACACAACCTCCATCTTCG  GTAAGAAACTGGTTCGCCCTCAT |
| LRPPRC | ATCCGACATGGTTACTGGTGGC  GTGTCAAGGACAGCAGATGAATC |
| HNRNPC | TCCTCCTCCTATTGCTCGGG  AGCCACTTTTGCCCCTTCG |
| HNRNPA2B1 | CAGCAACCTTCTAACTACGGTCC  CACTGCCTCCTGGACCATAGTT |
| IGF2BP1 | CTTTGTAGGGCGTCTCATTGGC  CCTTCACAGTGATGGTCCTCTC |
| IGF2BP2 | GTTGGTGCCATCATCGGAAAGG  TGGATGGTGACAGGCTTCTCTG |
| IGF2BP3 | TATATCGGAAACCTCAGCGAGA  GGACCGAGTGCTCAACTTCT |
| RBM15B | TGGTAACCTGGACCACAGCGTA  GGTTCTGGAACTTGAGGAAGGC |
| RBMX | TGGAAGCAGTCGCTATGATG  GAGGGTACCCCCTTTCCATA |
| METTL3 | CAACATACCCGTACTACAGGA  TTCATCTACCCGTTCATACCC |

**Supplementary Table 2:**

**Stemness primers used in RT-qPCR**

| **Genes** | **Primers** |
| --- | --- |
| CD133 | ACATGAAAAGACCTGGGGG  GATCTGGTGTCCCAGCATG |
| EPCAM | AATCGTCAATGCCAGTGTACTT  TCTCATCGCAGTCAGGATCATAA |
| CD24 | CTCCTACCCACGCAGATTTATTC  AGAGTGAGACCACGAAGAGAC |
| OCT4 | AGGCAACCTGGAGAATTTGTTC CACACTCGGACCACATCCTTC |
| NANOG | CAGGACAGCCCTGATTCTTCC TTTGCGACACTATTCTCTGCAGA |
| SOX2 | TACAGCATGTCCTACTCGCAG  GAGGAAGAGGTAACCACAGGG |
| ALDH | CTGCTGGCGACAATGGAGT CGCAATGTTTTGATGCAGCCT |
| CD44 | CTGCCGCTTTGCAGGTGTA CATTGTGGGCAAGGTGCTATT |
| GAPDH | GAAGATGGTGATGGGATTTC  GAAGGTGAAGGTCGGAGTC |
